# Gastric Outlet Obstruction from Stomach-Containing Groin Hernias: Case Report and a Systematic Review

**DOI:** 10.3390/jcm13010155

**Published:** 2023-12-27

**Authors:** Juan G. Favela, Madison B. Argo, Jared McAllister, Caitlyn L. Waldrop, Sergio Huerta

**Affiliations:** 1 Department of Surgery, University of Wisconsin Hospital and Clinics, Madison, WI 53792, USA; jfavela1192@gmail.com (J.G.F.); madisonargo14@gmail.com (M.B.A.); 2 Department of Surgery, VA North Texas Health Care System, Dallas, TX 75216, USA; jared.mcallister@va.gov (J.M.); caitlyn.waldrop@phhs.org (C.L.W.)

**Keywords:** femoral hernia, groin hernia, inguinal hernia, sliding hernia, gastric obstruction, gastric inguinal hernia

## Abstract

Most abdominopelvic structures can find their way to a groin hernia. However, location, and relative fixation are important for migration. Gastric outlet obstruction (GOO) from a stomach-containing groin hernia (SCOGH) is exceedingly rare. In the current report, we present a 77-year-old man who presented with GOO from SCOGH to our facility. We performed a review of the literature following the Preferred Reporting Items for Systematic Reviews and Meta-Analysis (PRISMA) of patients presenting with SCOGH since it was first reported in 1802. Ninety-one cases of SCOGH were identified (85 inguinal and six femoral) over the last two centuries (1802–2023). GOO from SCOGH occurred in 48% of patients in one review and 18% in our systematic analysis. Initial presentation ranged from a completely asymptomatic patient to peritonitis. Management varied from entirely conservative treatment to elective hernia repair to emergent laparotomy. Only one case of laparoscopic management was documented. Twenty-one deaths from SCOGH were reported, with most occurring in early manuscripts (1802–1896 [*n* = 9] and 1910–1997 [*n* = 10]). In the recent medical era, outcomes for patients with this rare clinical presentation are satisfactory and treatment ranging from conservative, non-operative management to surgical repair should be tailored towards patients’ clinical presentation.

## 1. Introduction

With more than 20 million groin hernia repairs performed every year worldwide, this intervention represents one of the most common operations performed by general surgeons globally [1,2]. It is not uncommon to find abdominopelvic organs within the groin hernia sac. Proximity, chronicity, and gravity make some organs more likely to be found within the femoral or inguinal hernia sac compared to others [3]. A chronic indirect inguinal hernia might lead to adherence of the posterior wall of the hernia sac to an intraabdominal viscus, making that wall indistinguishable from the hernia sac [4]. This type of hernia is called a sliding hernia [5]. Most commonly, the omentum, small bowel, and sigmoid colon are found on the left. On the right, ileocecal contents, or the bladder are often present in sliding hernias [5,6].

Less commonly, a Meckel’s diverticulum (a hernia of Littre) [7], the inflamed appendix (an Amyand hernia) [8], or ovaries with fallopian tubes [9] can be found within the hernia sac. Other eponyms in groin hernias include a two loop incarceration of the small bowel creating a “W” configuration, termed a Maydl’s hernia [10], and an inflamed appendix within the femoral canal, called a de Garengeot hernia [11].

Exceedingly rare contents of the hernia sac include the ureter [12], transverse colon [13], the pancreas [14], and the gallbladder [15]. The spleen [16] and the uterus [17] have also been documented in inguinal hernias in newborns with congenital disorders. The stomach is also uncommonly found in groin hernias.

The stomach is a fixed structure in the upper abdomen. The gastrophrenic, gastrosplenic, hepatogastric, and hepatoduodenal ligaments provide fixation to the stomach superiorly [18]. This arrangement combined with distance, makes the stomach an unusual visitor to the groin. The groin finds a visiting stomach in the following fashion. First, the inferior fixation of the stomach to the greater omentum and the gastrocolic ligament is more tenuous compared to the superior fixation. Initial migration of the omentum to the groin hernia sac with continuous traction and chronicity may eventually lead the stomach to travel within the groin hernia sac [19]. This downward movement of the stomach is termed gastroptosis [20].

Given the relative fixation and lack of proximity of the stomach to the groin, it is extremely rare to find it within the groin hernial contents. Thus, stomach-containing groin hernias (SCOGH) have uncommonly been reported in the literature with less than one hundred cases since it was initially documented in 1802 [21]. The clinical presentation of patients with SCOGH is highly variable but might include gastric outlet obstruction (GOO).

Given the earlier presentation of groin hernias in high-income countries (HICs) compared to low- to middle- income countries (LMICs) as well as the use of multiple diagnostic modalities such as computer tomography in HICs compared to LMICs, it would be less likely to find GOO from SCOGH in HICs [22]. The present report outlines a patient who presented to our institution with a long-standing groin hernia and GOO. The differential diagnosis included a small bowel obstruction from an incarcerated groin hernia, GOO from pectic ulcer disease or other causes of GOO such as a hiatal hernia.

In this report, we reviewed the literature of patients with SCOGH and present a case recently encountered in our practice. Pathophysiology, a brief history, and outcomes related to this rare entity are discussed.

## 2. Case Presentation

We review a case report of a patient who presented with a SCOGH at our institution. The medical records were reviewed for this patient in the computer patient record system. This work has been reported in line with the SCARE [23].

### Case Report

A 77-year-old man with chronic obstructive pulmonary disease (COPD; on 4 liters of oxygen), hypertension, and class III chronic kidney disease presented to the Emergency Department (ED) at our institution in February of 2023 with an incarcerated left inguinal hernia. He had a one-day history of abdominal pain, nausea, and vomiting. He reported presence of the hernia for over 10 years. His last bowel movement had been the night prior to presentation to the ED. On physical examination, he was tachycardic to 100 beats per minute and normotensive with a blood pressure of 110/70 mmHg. His abdomen was soft and mildly tender to deep palpation. There was no evidence of peritonitis. He had a left inguinal bulge that was tender to palpation and irreducible. He had no leukocytosis, and his serum lactic acid level was within normal limits. A surgery consult was placed by the ED with a differential diagnosis of a small bowel obstruction from an incarcerated groin hernia. A large bowel obstruction from an incarcerated groin hernia was also possible. He did not have a history of abdominal operations such that a small bowel obstruction from adhesion disease was less likely. Given the absence of peritonitis, a perforated viscous was also less likely.

**Figure 1:**
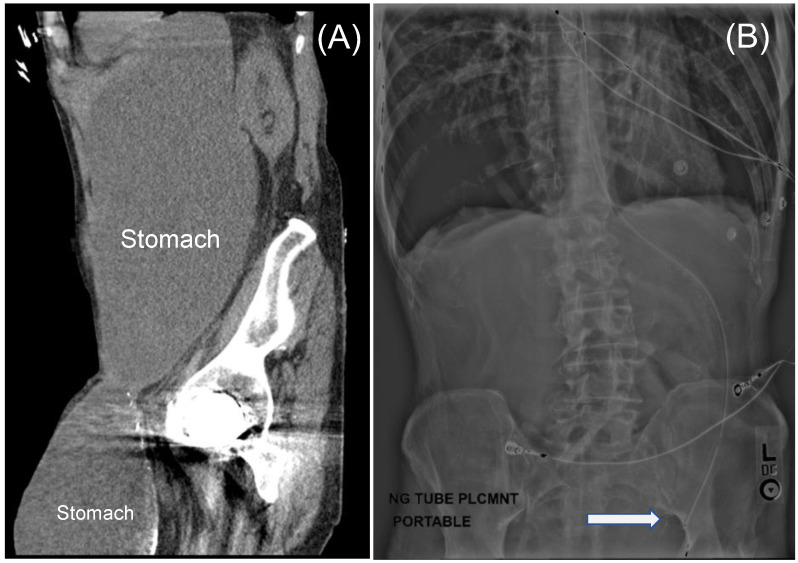
(**A**), Sagittal computed tomography image of the abdomen depicting the stomach extending into the left groin causing gastric outlet obstruction. (**B**), Coronal radiograph of the abdomen depicting the tip of the nasogastric tube in the left groin (arrow).

Due to the patient’s presentation and symptomatology, a nasogastric tube (NGT) was placed in the emergency department for decompression. A subsequent Kidney-Ureter-Bladder X-ray (KUB) demonstrated the tip of the NGT within the left groin. Computed tomography confirmed GOO from an incarcerated stomach within the left groin hernia. There was no radiographic evidence of bowel ischemia or compromise (Figure 1).

Intravenous fluid (IVF) administration and NGT decompression were immediately started. His tachycardia promptly resolved after initiating IVF. Serial abdominal examinations were performed and after a few hours of NGT decompression his left inguinal hernia was reduced. Because he was not interested in surgical intervention, he was started on oral feeds a day later and once he was tolerating his diet well, he was discharged home from the hospital. He was doing well at a six-week follow up visit in the clinic and still not interested in elective surgical repair of his hernia.

## 3. Review of the Literature

Given the usual presentation of GOO from SCOGH, a systematic review of the literature was undertaken following the Preferred Reporting Items for Systematic Reviews and Meta-Analysis (PRISMA) guidelines [23]. The primary outcome of this review was to identify the current incidence as reported in the literature of GOO from SCOGH. The secondary outcome was to determine clinical presentation of these patients, diagnostic modalities, and management as well as outcomes as they have occurred in history since this entity was originally described. This information was important in order to offer alternatives to the patient presented in this report.

SH, JF, and MA reviewed all the papers and selected all manuscripts required for inclusion. The initial literature review was performed on 21 March 2023. Various combinations of keywords including “hernia”, “inguinal hernia”, “sliding hernia”, “femoral hernia”, “groin”, and “stomach” were used for our searches. Gastric outlet obstruction from other causes was excluded from our review. No time restriction (beyond that of the existing databases) or language restriction was imposed. Databases including PubMed, MEDLINE (via PubMed), and Embase were initially queried. Subsequently, Cochrane Library, Google, Google Scholar, and ResearchGate were utilized to search and acquire reports that were new and/or unavailable from the previous databases. Further manuscripts were identified by close examination of the references of the index papers and main reviews on this subject [24,25,26]. These manuscripts were included in our review if they were appropriate references and did not duplicate our original findings of patients with SCOGH reviewed elsewhere. JF, MA, and SH reviewed all the abstracts from papers that contained inclusion criteria. JF, MA, and SH reviewed all articles that met including criteria.

**Figure 2:**
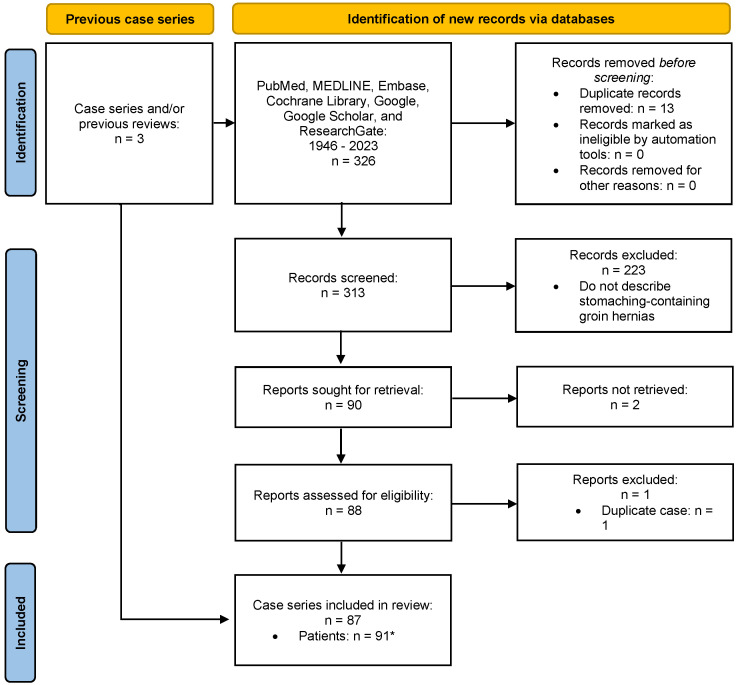
PRISMA flow-chart describing the process of our literature search. * Total number of patients includes our present case.

The PRISMA flow chart depicts the screening process (Figure 2). All the abstracts were analyzed within an EndNote group to eliminate irrelevant and duplicated studies. Google translate was utilized to translate articles in other languages. Full text for a handful of articles was unavailable for a variety of reasons. These include lack of electronic copies, restrictions by foreign countries, incomplete scanning, and older manuscript dates.

## 4. Results

### 4.1. Review of the Literature

A systematic review of the literature revealed 90 cases of SCOGH, with the present report adding an additional case to the world literature. Other than English, the Spanish and French literature were the most reported languages identifying this clinical entity. Most reports include single cases with a literature review at the time of the publication. Several documents indicated the existence of only 60 cases prior to 1980 [26,27,28].

The first comprehensive review of SCOGH was published by Davey and Strange in 1954 [25]. This manuscript accounted for 34 inguinal and three femoral hernias and was inclusive of the prior 150 years and up to the date of publication [25]. A second review in 1960 added only 6 further cases of inguinal hernias with stomach contents to the literature [26].

The most recent review includes 21 cases from 1942 to 2020 of patients who presented with SCOGH [24]. Within this review, there were 10 patients who had GOO. This manuscript focused on the management of this condition with emphasis on patients presenting acutely because of perforation. This review was of the English literature only and was limited to reports digitally available. All patients with gastric perforation required laparotomy with one exception, which was addressed laparoscopically [24]. Our analysis identified 90 unique patients with SCOGH encompassing a period of over two centuries (1802 to 2022). In addition, we include a patient who presented to our institution in February of 2023 with GOO from SCOGH. Thus, a total of 91 patients (85 inguinal and 6 femoral) are included in the present review (Table 1). If available, patient characteristics and clinical presentation for each case are included in Table 2 and Table 3, inguinal and femoral cases, respectively.

#### 4.1.1. History

The first case of a hernia sac containing stomach appeared in the literature in 1802 [21], and it was diagnosed at autopsy. The patient had been suffering from symptoms of this hernia for 32 years until his demise at age 64 [21]. It took 152 years for the first comprehensive and intriguing review to appear in the literature by Davey and Strange in 1954 [25]. This review included 34 inguinal and three femoral hernias. Our review adds 51 inguinal (including one of our own) and three femoral hernias to the world literature.

The first eight reports of SCOGH were documented at autopsy (*n* = 8 from 1802 to 1896) [21]. The first case of a femoral hernia containing stomach was documented by Keller in 1885 [29]. The first laparotomy performed identifying SCOGH occurred in 1897 [30]. The first radiographic evidence of SCOGH was first published in the literature in 1915 [30]. Successful outcomes for an emergent operation from SCOGH were initially described by Elischer in 1923 [31]. The first woman with SCOGH was reported in 1925 [25,32]. The first case of SCOGH repaired in the elective setting is credited to de Vernejoul and de Luna in 1925 [32].

#### 4.1.2. Mechanism of Migration

Multiple mechanisms for the descent of the stomach into the groin have been proposed as early as 1912 by Chambard, [19] 1927 by Sicot, [33] and 1930 by Novaro [34]. Three mechanisms remain constant in the literature: (1) downward pulling of the omentum into the inguinal hernia sac; (2) chronicity [this is evidenced by our review, which found that the average of time of a hernia in patients with SCOGH was 23.1 ± 10.8 years (range 10 to 50 years)]; and (3) short stature of patients. Downward deviation of the diaphragm as a result of chronic COPD might also be a contributing factor [27]. Giant inguinoscrotal hernias, defined by the extension of the hernia down to midthigh while the patient is standing, has also been documented as a risk factor [35]. The likelihood of all mechanisms occurring in groin hernias simultaneously leading to SCOGH is so infrequent that in over more than two centuries less than 100 stomach-containing groin hernias have been reported in the literature.

#### 4.1.3. Patient Demographics

The mean age for the entire cohort was 69.6 ± 12.9 years-old (range 28 to 87 years-old) (Table 1). Most patients were men overall, but female gender was more common in femoral hernias with SCOGH (66.7%). For 73 cases, laterality was included in the reports and 97.5% occurred on the left. Two cases reported bilateral hernias. Six patients had femoral hernias.

**Table 1:** Characteristics of patients presenting with stomach-containing groin hernias (*n* = 91).

Characteristics	Inguinal (*n* = 85)	Femoral (*n* = 6)
Age [Years (SD) *]	74.2 (13.0)	62.0 (11.5)
Sex [male (%)]	95.2	33.3
Laterality [Left (%)]	78.0	100.0

* Standard Deviation.

#### 4.1.4. Management

Nine inguinal and one femoral hernia containing stomach were reported at autopsy. These were all early reports (1802 to 1896). Elischer successfully operated emergently on two patients with SCOGH in 1923 [31]. The first hernia repaired in the elective setting was reported by de Vernejoul and de Luna in 1925 [32].

Heylen’s manuscript addressed the management of this condition with emphasis on patients presenting acutely because of gastric perforation. All patients with gastric perforation required laparotomy with one exception, which was addressed laparoscopically [24].

Conservative management was explicitly undertaken in 12 patients with SCOGH. The reasons for this approach were cited as high-risk operative candidates. Nasogastric decompression was initially undertaken in patients who presented with emesis or acute incarceration.

#### 4.1.5. Complications

The mean duration for history of a hernia was 23.1 ± 10.8 years (range 10 to 50 years). Gastrointestinal symptoms related to obstruction (nausea, emesis, abdominal pain) were the most commonly reported symptoms (47.8%). GOO was reported in 17, absent in 14, and not reported in 61 patients. Five cases explicitly reported no symptoms. Gastric rupture was emphasized for some reports and in a manuscript review [24,36]. Other complications included aspiration pneumonia directly attributed to this entity [37]. Gastric volvulus in a patient with SCOGH was reported in one manuscript [38].

#### 4.1.6. Mortality

Death directly attributed to SCOGH was reported in 21 cases (22.8%). However, most mortalities occurred in early publications (1802–1896 [*n* = 9] and 1910–1997 [*n* = 10]). Only two mortalities occurred in the recent era (2019 and 2021), but these patients were 75 and 84-years old, respectively. Only one death was identified from a femoral hernia containing stomach in a 47-year-old woman, which was reported in 1885 [29] (Table 2 and Table 3).

**Table 2:** Characteristics and clinical presentation for the 85 patients with stomach-containing inguinal hernias, ordered by chronological order of appearance in the literature.

*n*	Reference, Year	Age	Sex	Laterality	Clinical Presentation
1	Lallement, 1802 [21]	64	Male	NR	Abdominal pain/discomfort and vomiting
2	Yvan, 1830 [39]	NR	Male	NR	Vomiting
3	Febre, 1832 [40]	73	Male	Right	No symptoms
4	Fogt, 1884 [41]	60	Male	Left	Vomiting
5	Schmidt, 1885 [42]	65	Male	Left	Hematemesis and inguinal pain
6	Chiari, 1888 [43]	74	Male	Right	No symptoms
7	Lewin, 1893 [44]	53	Male	Left	Emesis and pain
8	Chevereau, 1894 [45]	77	Male	Left	Emesis and pain
9	Souligoux, 1896 [46]	NR	Male	Left	NR
10	Brunner, 1897 [30]	28	Male	NR	NR
11	Hilgeneriner, 1910 [47]	52	Female	Left	Pain and vomiting
12	Chambard, 1912 [19]	62	Male	Left	Vomiting, pain, and an incarcerated hernia
13	Rieder, 1915 [48]	62	Male	Left	Hematemesis and melena
14	Ahrens, 1920 [49]	40	Male	Right	Pain
15	Maag, 1920 [50]	81	Male	Left	No symptoms
16	Stokes, 1922 [51]	42	Male	Right	Vomiting and an incarcerated hernia
17	Elischer, 1923 [31]	53	Male	Left	Nausea and an incarcerated hernia
18	Elischer, 1923 [31]	70	Male	Left	Incarcerated hernia
19	Dressen, 1925 [52]	62	Male	Left	Vomiting, pain, and inguinal symptoms when eating
20	de Vernejoul, 1925 [32]	57	Female	Left	NR
21	Sicot, 1927 [33]	59	Male	Left	Pain, vomiting, and dyspepsia
22	Lipkin, 1928 [53]	60	Male	Left	Incarcerated hernia
23	Siegmund, 1929 [54]	NR	Male	Right	NR
24	Novaro, 1930 [34]	53	Male	Right	Vomiting, pain, and an irreducible hernia
25	Rodzevich, 1935 [55]	54	Male	Left	Vomiting and abdominal pain
26	Oakley, 1937 [56]	81	Male	Right	Abdominal and groin pain
27	Herrmann, 1937 [57]	80	Male	Left	Vomiting
28	Lemaitre, 1937 [58]	51	Male	Left	Dyspepsia
29	Lust, 1937 [59]	62	Male	Left	NR
30	Alexsandrovskiv, 1940 [60]	73	Male	Left	Incarcerated hernia
31	Feldman, 1943 [61]	66	Male	Right	No symptoms
32	Hartley, 1945 [62]	67	Male	Left	Dyspepsia
33	Simmons, 1949 [63]	66	Male	Left	Nausea, vomiting, and abdominal pain
34	Lewis, 1950 [64]	69	Male	Right	Occasional vomiting
35	Anger, 1952 [65]	74	Male	Left	Vague symptoms
36	Bernard, 1953 [66]	NR	NR	NR	NR
37	Meinterz, 1953 [67]	NR	NR	NR	NR
38	Davey, 1954 [25]	61	Male	Left	Vomiting with markedly distended and tense abdomen
39	Legrand, 1955 [68]	NR	NR	NR	NR
40	D’Eshougues, 1956 [69]	NR	NR	NR	NR
41	Allende, 1956 [70]	NR	NR	NR	NR
42	Kislenskii, 1959 [71]	NR	NR	Left	NR
43	Hagarty, 1959 [72]	NR	NR	NR	NR
44	Ship, 1960 [26]	83	Male	Left	Persistent nausea and vomiting
45	Herrera, 1960 [73]	NR	NR	NR	NR
46	Jackson, 1964 [74]	NR	NR	NR	Strangulation and perforation of the stomach in the inguinal canal
47	Falugiani, 1968 ^a^	NR	NR	NR	NR
48	Gue, 1970 [75]	NR	NR	NR	NR
49	Soudek, 1975 [76]	NR	NR	NR	NR
50	Padmanabhan, 1976 [77]	65	Male	Left	NR
51	Nagendran, 1977 [78]	NR	NR	NR	NR
52	Rozencwajg, 1981 [79]	NR	NR	NR	NR
53	Udwadia, 1984 [80]	NR	NR	NR	Hematemesis
54	Quaranta, 1984 [81]	NR	NR	NR	NR
55	Resente,1986 [82]	NR	NR	NR	NR
56	Naraynsingh, 1987 [83]	62	Male	Left	Recurrent bouts of vomiting, recurrent GOO
57	Levy, 1987 [84]	49	Male	Left	Abdominal pain, nausea, and weight loss
58	Loizate, 1988 [85]	NR	NR	NR	Upper gastrointestinal tract hemorrhage
59	Broquet, 1992 [86]	64	Female	Bilateral	Perforation of gastric ulcer within the hernia sac
60	Diaz, 1997 [27]	NR	NR	NR	NR
61	Diaz, 1997 [27]	NR	NR	NR	NR
62	Walgenbach, 2001 [87]	72	Male	Left	A 6-h history of abdominal distension and pain
63	Birnbaum, 2011 [88]	86	Male	Right	Nausea and vomiting
64	Dogar, 2011 [89]	65	Male	Left	Irreducible groin bulge, abdominal pain, distention, darkish red vomitus, and obstipation
65	Kerschaever, 2012 [90]	79	Male	Left	Anorexia, vomiting, and abdominal distension
66	Ogul, 2013 [28]	56	Male	Left	Recurrent vomiting and bilateral incarcerated groin bulges
67	Ferdinand, 2013 [38]	73	Male	Right	Iron deficiency anemia and gastric volvulus
68	Fazekas, 2014 [37]	85	Male	Left	Three-day history of gastrointestinal obstructive symptoms
69	Creedon, 2014 [91]	87	Male	Left	Colicky abdominal pain for 48 h and vomiting
70	Patel, 2014 [92]	85	Male	Left	3-day history of profuse vomiting and abdominal pain
71	Lajevardi, 2015 [35]	83	Male	Left	Four-day history of vomiting and constipation
72	Fitz, 2016 [14]	46	Male	Bilateral	Severe abdominal pain after dinner brought in by ambulance to the emergency department
73	Mora-Guzman, 2016 [93]	79	Male	Right	Abdominal pain and vomiting
74	Periz-Pueyo, 2016 [94]	61	Male	Left	Gastric necrosis secondary to an incarcerated inguinal hernia
75	Nugud, 2017 [95]	67	Male	Left	Bilious vomiting with abdominal pain
76	Sayad, 2019 [96]	50	Male	NR	Severe abdominal pain
77	Junge, 2019 [97]	75	Male	Left	Abdominal pain and nausea
78	Mehta, 2019 [98]	75	Male	Left	5-day history of hematemesis
79	Heylen, 2020 [36]	74	Male	Left	Dark vomitus and generalized abdominal tenderness
80	Patel, 2021 ^a^	84	Male	NR	Nausea, vomiting, constipation, GOO, peritonitis
81	Vinod, 2021 [99]	49	Male	Left	Acute abdominal pain with nausea and dysuria
82	Alexandre, 2022 [100]	71	Male	Left	Nausea, vomiting, constipation, and GOO
83	Grantham, 2022 [18]	81	Male	Lett	Coffee ground emesis
84	Abbakar, 2022 [101]	84	Male	Right	Double GOO, abdominal pain and vomiting
85	Huerta, 2023 ^a^	77	Male	Left	Abdominal pain, nausea, vomiting, and GOO

GOO = gastric outlet obstruction; NR = not recorded; ^a^ From posters, abstracts, presentations, and/or this research.

**Table 3:** Characteristics and clinical presentation for the 6 patients with stomach-containing femoral hernias, ordered by chronological order of appearance in the literature.

Reference, Year	Age	Sex	Laterality	Clinical Presentation
Keller, 1885 [29]	47	Female	Left	Abdominal pain and vomiting
Spiegel, 1920 [102]	55	Female	Left	Gastric strangulation
Cave, 1948 [103]	56	Female	Left	Dyspepsia
Davey, 1954 [25]	68	Male	Left	No symptoms
Cade, 1984 [104]	79	Female	Left	Abdominal pain, emesis, and hematemesis
Natsis, 2008 [20]	67	Male	Left	Findings at autopsy

## 5. Discussion

The present report discusses an unusual hernia in a HIC that was neglected for over 10 years and was not suspected of being stomach containing until the patient presented with GOO to our institution. The senior author of the present manuscript (SH) has performed nearly 2000 groin hernias in HICs and LMICs for the past twenty-years, and this is the first case of a SCOGH. Given the unusual finding of the hernia containing stomach, we performed a literature review to determine the incidence, natural history, and management of this conditions such that treatment options including observation could be offer to the patient that presented to our institution. This review might provide recommendations for surgeons encountering this uncommon type of hernia, especially in LMICs.

We offer the most comprehensive review of the literature today. Our review showed that the English, Spanish, and French literature were the dominant entities in publishing these reports. However, we have previously shown that emergent groin hernias are more commonly encountered in LMICs compared to HICs [22]. Thus, HICs that are less likely to encounter this entity due to earlier presentation of patients and more available diagnostic modalities are more likely to report it in the literature. The present data may serve as a staging platform for other surgeons encountering this entity to understand the incidence, natural history, and management of GOO from SCOGH. It is crucial to have this information available for surgeons in LMICs. Thus, open publications of GOO from SCOGH are essential.

Given our review of the literature, in case we encountered at our institution, the following elements were likely to play a role in the migration of the stomach to the groin: (1) chronicity [over ten years], (2) COPD, and (3) a giant inguinoscrotal hernia. But the differential diagnosis in the present case was a bowel obstruction (small or large) from an incarcerated hernia. While other diagnoses such as GOO from large hiatal hernia or adhesions would be possible, given the presence of an incarcerated groin hernia in the present report, excluded other possibilities. A finding of the NGT within the groin by KUB was diagnostic for this patient but it was confirmed by computed tomography.

Laterality and gender are important for migration of the stomach into the groin. Our analysis identified left laterality for 78% and 100% of the cases and male gender for 95% and 33% of the cases for inguinal and femoral hernias in patients with SCOGH, respectively. These findings are consistent with the patient encountered at our institution (a man with a long history of a left inguinal hernia).

Our review of the literature showed a clinical presentation for SCOGH that ranged from entirely asymptomatic to an acute abdomen. Overall, the most common complaint was related to obstructive symptoms, occurring in nearly half of the cases in our analysis. GOO from SCOGH occurred in 18.5% of cases. Thus, the present case adds a report to the world literature. It was important to review the literature to determine treatment options that could be offered to the patient in the present report.

Our review showed a wide spectrum of management strategies in patients with SCOGH from entirely conservative (*n* = 11; 12%), to elective operative intervention, to emergent operative management. Management varied from a groin approach with or without laparotomy for elective cases to laparotomy with or without a groin incision in emergent cases. One report documented an exclusively laparoscopic hernia repair [24]. Haylen’s manuscript provides an excellent review of the surgical approach depending on clinical presentation [24]. Thus, our review was important as it showed the permissibility of managing some of these patients conservatively. The patient in the present report elected not to undergo surgical intervention, but elective repair was offered both while he was in the hospital and during a follow up clinic appointment.

Given the case we encountered at our institution and our review of the literature, we found that GOO from SCOGH requires a high degree of suspicion. Inguinoscrotal hernias with a great deal of chronicity might raise suspicion of SCOGH. History and a physical exam should guide the differential diagnosis and exclude other forms of bowel obstruction leading to GOO. Computed tomography is definitive for the diagnosis. However, in the absence of tomography an NGT found in the groin by KUB should be diagnostic. Ultrasonography might also be helpful. In the absence of peritonitis, patients should be treated with bowel decompression and intravenous hydration. Elective repair via the posterior or anterior approach can be offered for patients depending on the comfort of the surgeon and on the surgical risk of the patient.

There are several limitations with this study. One of the most substantial limitations of our retrospective review emanates from the inability to accurately retrieve all the data from the medical charts. However, this is a limitation of all reviews of the literature. Another limitation of this study has to do with the references for each of the cases presented in our review. Several references were from older manuscripts and the initial citation was extracted from the reference list of the reviews included in this paper. However, they do not contain all the information that current reference databases extract for appropriately identifying these references in scholarly websites. However, as it is, the present manuscript should represent the best referenced manuscript for patients with SCOGH. Our review of the literature is limited by the likelihood that this condition is underreported. The underreporting of this entity is more likely to occur in LMICs where the incidence of GOO from SCOGH might be higher. Thus, publication bias is a highly likely limitation of this study. We should encourage authors from LMICs to publish their experience with unusual hernias to understand the true incidence of SCOGH.

## 6. Conclusions

Our review shows that GOO from SCOGH is uncommon and requires a high degree of suspicion. As in the present case report, patients might show symptoms related to GOO, though some patients may remain entirely asymptomatic as demonstrated in our review of the literature. Our patient elected not to proceed with surgical intervention and conservative management is permissible depending on clinical presentation, patient preferences, and operative risk. For SCOGH presenting with symptoms of GOO, immediate NGT decompression is encouraged and may allow for reduction of an initially incarcerated SCOGH and ultimately permit conservative or elective management. Modern diagnostic tools and contemporary management strategies allow for early identification and improved outcomes for patients with SCOGH.

## Data Availability

All data presented within this manuscript is available as described in the methods section.
